# Inflammatory nociception responses do not vary with age, but diminish with the pain history

**DOI:** 10.3389/fnagi.2014.00181

**Published:** 2014-07-28

**Authors:** Karina Simón-Arceo, Bernardo Contreras, Martha León-Olea, Ulises Coffeen, Orlando Jaimes, Francisco Pellicer

**Affiliations:** ^1^Laboratorio de Neurofisiología Integrativa, Dirección de Investigaciones en Neurociencias, Instituto Nacional de Psiquiatría Ramón de la Fuente MuñizTlalpan, México, D.F., México; ^2^Departamento de Neuromorfología Funcional, Dirección de Investigaciones en Neurociencias, Instituto Nacional de Psiquiatría Ramón de la Fuente MuñizTlalpan, México, D.F., México

**Keywords:** old age, nociception, inflammation, rat, pain

## Abstract

Some of the relevant factors that must be considered when dealing with old age include its growing numbers in the general population and pain contention in this age group. In this sense, it is important to study whether antinociceptive responses change with age. To elucidate this point, persistent pain in animals is the preferred model. In addition, the response to inflammatory pain in the same individual must be explored along its lifetime. Male Wistar rats were infiltrated with carrageenan (50 μl intraplantar) and tested 3 h and 24 h after injection using thermal (plantar test) and mechanociceptive tests (von Frey). The rats were divided into the following groups: (a) young rats infiltrated for the first time at 12 weeks of age and re-infiltrated at 15 and 17 weeks; (b) adult rats infiltrated for the first time at 28 weeks of age and re-infiltrated at 44 and 56 weeks; and (c) old rats infiltrated for the first time at 56 weeks of age and re-infiltrated at 72 weeks. The rats tested for the first time at 12 and 56 weeks of age showed hyperalgesia due to carrageenan infiltration at 3 h and 24 h after injection. This result showed that old rats maintain the same antialgesic response due to inflammation. However, when the injection was repeated in the three age groups, the latency to the thermal and mechanociceptive responses at 3 h is increased when compared to animals exposed for the first time to inflammation. The response to thermal and mechanociception in old rats is the same as in young animals as long as the nociceptive stimulus is not repeated. The repetition of the stimulus produces changes compatible with desensitization of the response and evidences the significance of algesic stimulus repetition in the same individual rather than the age of the individual.

## Introduction

One of the most vulnerable and complex groups in the management and treatment of pain is the elderly. The pain condition in this group is highly incapacitating since pain hinders the integration to daily social activities and the preservation of individual independence. Chronic pain is the most relevant issue in public health worldwide. This condition is present in 30–33% of the general population, notwithstanding the economic development of the country (Elzahaf et al., 2012). This problem gains importance given that the population pyramid is currently tending to revert, that is, the percentage of old people is increasing with time (World Health Organization, 2011).

Data concerning pain perception is controversial in the elderly compared to the young. The studies have been carried out in transverse samples in which the “pain history” or the type of damage that the subject has endured throughout his lifetime have not been taken into consideration (Ferrell, 1999; Gibson and Farrell, 2004; Helme et al., 2004; Farrell et al., 2005; Lautenbacher et al., 2005; Suokas et al., 2012).

Our altricial condition endows us with a nervous system that is not fully developed at birth. Therefore, many of our functions, especially those associated with perception, among which pain lies as an alarm system, are integrated along time. This adds a plastic factor which depends on the development of the individual. Aging is associated with a decline in several sensory modalities including nociception (Robertson and Grant, 1985; Bergman and Ulfhake, 1998). There is controversy with regard to the microanatomic constitution of the nervous system along the lifetime of experimental animals and humans. The most important characteristics that determine changes in the old axons are morphological changes in receptors, loss of peripheral sensory endings, changes in myelin and axonal injury related tropism. These changes modify the acquisition of the attributes of the sensory modalities, including those related to nociception (Bergman and Ulfhake, 2002).

A large variety of antialgesic responses have been reported in aged rodents with inconclusive results. These may be due to the different strains of rats, mice and also guinea pigs used in the experiments. Some of these strains possess special characteristics such as the Lou/C/Jall rats (Jourdan et al., 2000), Fischer 344/DuCrj mice (Iwata et al., 2002), C57BL/6NIA (B6) mice (Wang et al., 2006) and guinea pigs (McDougall and Schuelert, 2007). Furthermore, the wide range of ages in aging rats, which goes from 14 months old to 30 raises difficulties in the interpretation of the results.

Harkins (1996) have even proposed the term “presbyalgos” to denote the sensory impairment of pain in old age analogous with the loss of vision “presbyopia” at this stage of life. Given the complexity of the approach to pain perception and response in old age and through lifetime, we decided to analyze this issue in rats using experiments in which the type, intensity, and duration of pain could be controlled.

The aim of this study was to determine the changes of the thermal and mechanociceptive responses, under controlled experimental parameters such as stimulus intensity, duration, and periodicity in a carrageenan inflammatory pain model, at different ages (young, adult and old rats) and when the nociceptive stimulus is repeated in the same individual along its lifetime.

## Methods

Experiments were conducted in compliance with the guidelines of the Ethics Committee of the International Association for the Study of Pain (Zimmerman, 1983) and with the approval of the Projects and Bioethics Commission of our institution (NC093230.0).

Male Wistar rats of the following ages were used in the experiments: 12 weeks (260 ± 36.2 g, *n* = 12), 28 weeks (467 ± 16.5 g, *n* = 9) and 56 weeks (550 ± 24.3 g, *n* = 10). Rats were raised, housed and maintained at our own facilities. During the observation period, they were kept in transparent acrylic cages, under laboratory conditions with a 12 h:12 h light/dark cycle at 23°C and with food and water *ad libitum*. To diminish stress, experimental animals were submitted to a habituation period in the experimental acrylic cage, which consisted of 20-min sessions for three consecutive days. On the fourth day, experiments were carried out.

### Inflammatory procedure and inflammation measurement

Prior to the injection, rats were kept inside an acrylic cage and anesthetized by the inhalation of a mixture of isoflurane (2%) and oxygen (98%). The inflammatory process was induced by the intraplantar injection of carrageenan lambda [Sigma Chemical Co., St. Louis, MO, USA; 1% carrageenan (CAR) in saline solution, 50 μl] into the right hind paw. The plantar perimeter of the right hind paw was measured at the metatarsal level with a cotton thread to the nearest millimeter prior to the CAR intraplantar injection, as well as at 3 and 24 h post-CAR injection (López-Avila et al., 2004; de Rienzo-Madero et al., 2013).

### Nociceptive behavior

The thermonociceptive response was elicited by applying punctual radiant heat in the Plantar Test Apparatus (Ugo Basile, mod 7370) according to the Hargreaves Method. Paw withdrawal latency (PWL) was determined for the right paw to the nearest 0.1 s using the electronic timer in the device. Cut-off time was 20 s to avoid tissue injury. Each determination was made with the average of three trials per hind paw. All groups were tested prior to the induction of inflammation to determine the nociceptive threshold (acute pain). PWL was also measured 3 h and 24 h following CAR intraplantar injection.

The mechanociceptive response was elicited with a graduate pressure monofilament tip in a Dynamic Plantar Aesthesiometer (Ugo Basile, mod 37400-001). The monofilament can progressively increase force from 0 to 50 g (10 s ramp). The paw withdrawal threshold (PWT) was determined to the nearest 0.1 s and 0.5 g of force using the electronic timer in the device. Each determination was made with the average of five trials. All groups were tested prior to the induction of inflammation to determine the nociceptive threshold (acute pain). PWT was also measured 3 and 24 h following intraplantar CAR injection.

### Experimental groups

Animal groups were divided as follows: (a) young rats tested at 12 weeks of age and subsequently at 15 and 17 weeks; (b) adult rats tested for the first time at 28 weeks and re-infiltrated at 44 and 56 weeks old; and (c) old rats first tested at 56 weeks old and subsequently at 72 weeks of age. At the end of the experiments, animals were sacrificed with a sodium pentobarbital overdose.

### Histology and paw inflammation

Rats (*n* = 4) were deeply anesthetized with sodium pentobarbital and were perfused intracardially with clearing solution (150–200 ml of 0.9% saline with heparin 2500 UI/500 ml, followed by 350–400 ml of 4% paraformaldehyde (Sigma Chemical Co., St. Louis, MO) in PBS (0.1 M phosphate buffer saline, pH 7.4). Controls and inflammated paws were removed and post fixed for 12 h in same fixative at 4°C, after which they were cryoprotected in 30% sucrose and stored at 4°C until used. Paws were dissected and cut at 15 μm in a cryostat microtome and mounted on previously gelatinized glass slides.

For Nissl staining, slices were immersed in (0.5%) cresyl violet solution containing: sodium acetate (2.7%), acetic glacial acid (0.92%) in 100 ml for 2 min. Sections were dehydrated through a series of graded ethyl alcohols (70, 80 and 96% for 2 min each and 100% twice for 2 min), cleared in xylene twice (5 min) and cover-slipped with Entellan resin.

### Statistical analysis

Differences between average PWL and PWT values in control conditions, 3 h and 24 h after CAR injection in young, adult, and old rats were assessed by ANOVA and a *post hoc* Holms Sidak test (*p* ≤ 0.05). Similarly, PWL and PWT values from the first exposure to the inflammatory stimulus were compared, as well as the subsequent values in the three groups (young, adult and old).

## Results

**Figure 1:**
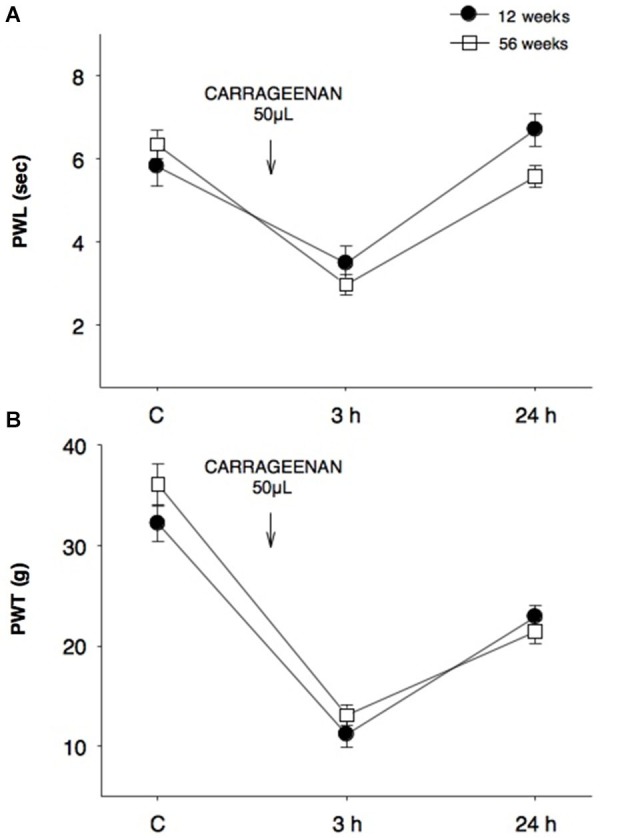
**Comparison of thermal and mechanociceptive responses between young and old rats.** **(A)** Paw withdrawal latency (PWL, s) and **(B)** paw withdrawal threshold (PWT, g) values in 12-week old young rats (black dots) and 56-week old rats (white squares) in control (C), 3 and 24 h after CAR intraplantar injection for the first time. No significant differences were found between groups. Data are expressed as mean ± SE.

**Figure 2:**
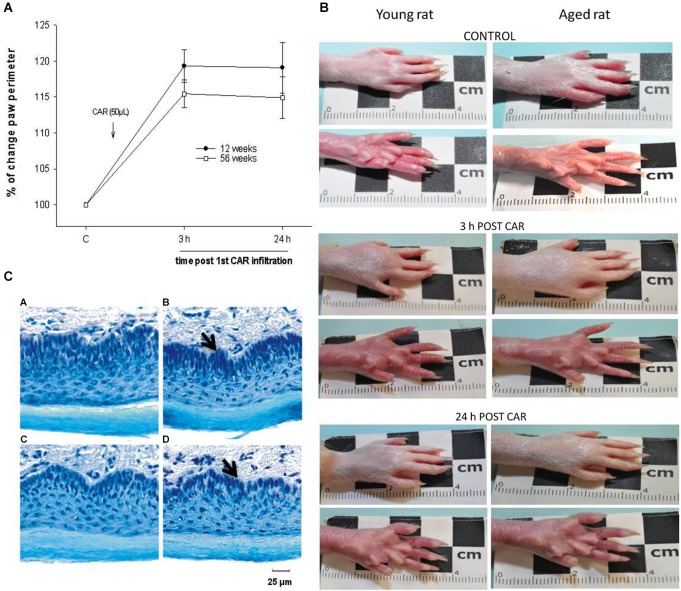
**(A)** The percentage of change in plantar perimeter produced by CAR infiltration in young (12 week-old, black dots) and aged (56 week-old, white squares) rats is shown. Note that at 3 and 24 h posterior to infiltration there are no significant changes between groups (*F* = 2.404; *p* = 0.109). **(B)** Photographs showing temporal course of the inflammatory process induced by CAR infiltration in young and aged rats. Note the similarity between plantar inflammation after 3 and 24 h of CAR application in young and aged rats. **(C)** Photomicrography’s of tissue sections of young and aged rat paws (15 μm thick), using the Nissl technique. The figure shows the similarities in paw cytoarchitecture of young (panel **C**, A), CAR-infiltrated young (panel **C**, B), aged (panel **C**, C) and CAR-infiltrated aged (panel **C**, D) rats. CAR injection was applied into the right paw. Notice the epidermal layer in the bottom of each photomicrography. Panel **C**, B and D show a compression of the internal dermal layer related to the inflamed connective tissue (arrow). Of relevance are the preservation of the dimensions of the layers and the cytoarchitecture.

Our results showed that the nociceptive process induced by inflammation did not yield significant differences in young (12 weeks) and old (56 weeks) rats when assayed in thermal (Figure 1A) and mechanociceptive (Figure 1B) trials, when the stimulus was given for the first time. The responses were similar in the control situation, as well as 3 and 24 h posterior to the induction of the inflammatory process. The edema produced by the first CAR administration was measured in young and aged rats, and no changes were observed between groups (Figure 2).

**Figure 3:**
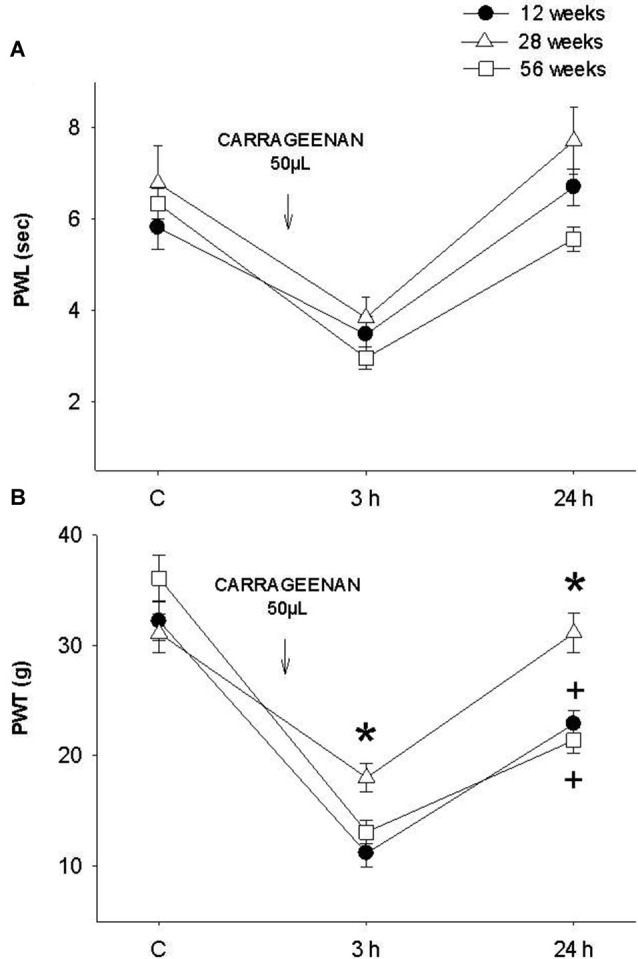
**(A)** Thermal and **(B)** mechanociceptive responses in young (12 weeks old, black dots), adult (28 weeks old, white triangles) and old (56 weeks of age, white squares) rats in control, 3 h and 24 h after the first application of intraplantar CAR. In the mechanociceptive test **(B)** adult rats present average values significantly higher than those of young and old rats at 3 h (*F* = 10.327, * *p* = 0.001) and 24 h (*F* = 12.403, * *p* = 0.001) posterior to inflammation. Notice that the values of young and old rats were significantly smaller at 24 h and different to those of the adult group (+ *p* = 0.01). Data are expressed as mean ± SE.

When comparing the young rat group (12 weeks) with adult (28 weeks) and old (56 weeks) animals exposed for the first time to the inflammatory process, no significant differences were recorded in PWL (*F* = 3.122, *p* = 0.06, Figure 3A). Alternatively, in the mechanociceptive test at 3 h and 24 h, 28-week old rats presented a significantly greater PWT than 12- and 56-week old animals (*F* = 10.327, *p* = 0.001 (3 h) and *F* = 12.403, *p* = 0.001 (24 h), Figure 3B). It is important to mention that 24 h after infiltration the mean values of adult rats returned to their control values. In contrast, values of young and old rats were significantly smaller at 24 h and different to those of the adult group.

**Figure 4:**
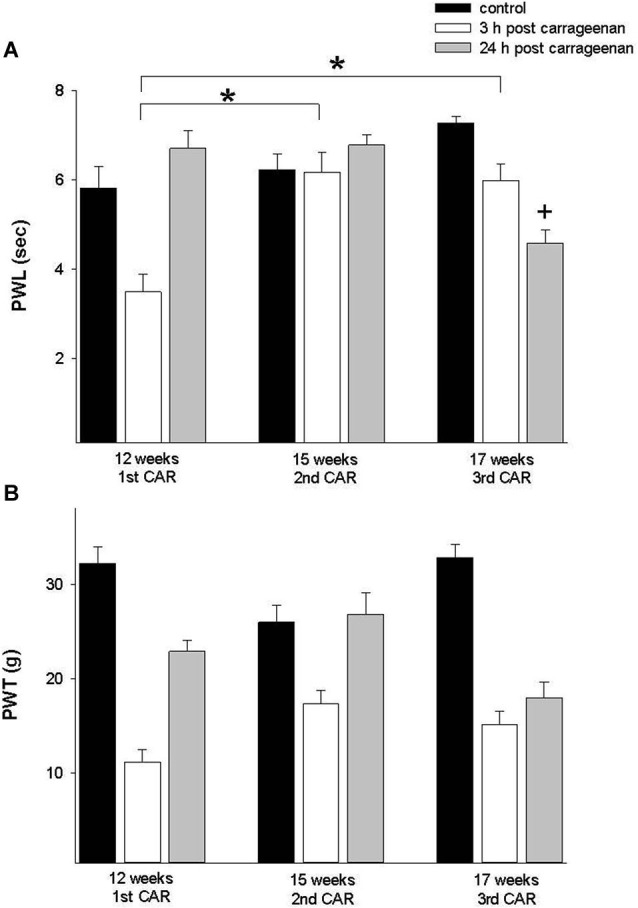
**(A)** Effect of a first time CAR infiltration at 12 weeks old, and repeated CAR application at 15 and 17 weeks of age. Notice a significant increase in PWL 3 h after injection (white bars) in comparison with mean values obtained at 12 weeks (* *p* = 0.01). **(B)** Similar changes are observed in PWT at 12 and 15 weeks. Data are expressed as mean ± SE.

To determine the effect of the repetition of the inflammatory process in the same individual at different ages, we used young animals assayed in the thermal and mechanociceptive trials at 12 weeks of age and subsequently the inflammatory process was repeated at 15 and 17 weeks old. The results showed a significant increase in the latency of thermonociceptive response at 3 and 24 h post CAR injection (PWL; *F* = 10.696, *p* = 0 0.001, Figure 4). The mechanociceptive response did not show statistical significance.

**Figure 5:**
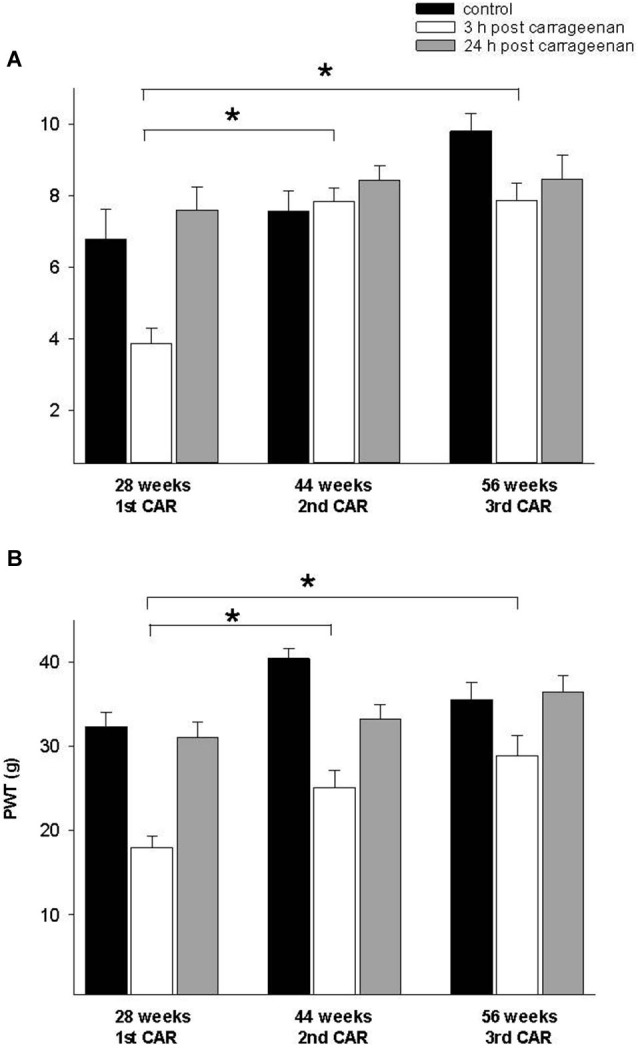
**(A)** Effect of a first time CAR infiltration at 28 weeks old, and repeated CAR application at 44 and 56 weeks of age. Notice that 2nd and 3rd CAR application at 44 and 56 weeks of age caused a significant increase in PWL 3 h after injection (white bars) in comparison with mean values obtained at 28 weeks. **(B)** Similar changes are observed in PWT (* *p* = 0.01). Notice the race in control mean values at 44 and 56 weeks old rats before the second and third repetition of CAR infiltration. Data are expressed as mean ± SE.

**Figure 6:**
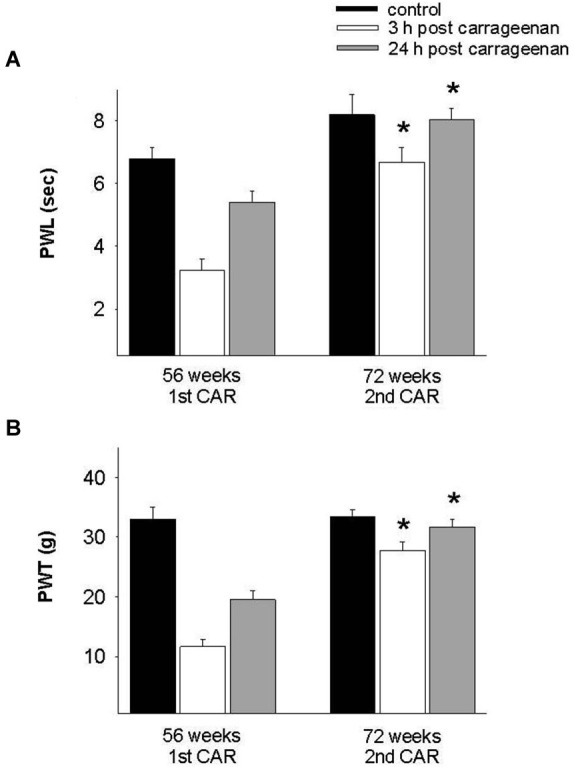
**Effect of repeated inflammation on nociceptive responses in old rats at 72 weeks of age. (A)** In old rats, repeated CAR application at 72 weeks old causes a significant increase in PWL 3 h post-injection (white bars) as compared with values obtained after the first injection at 56 weeks (*F* = 17.829, *p* = 0.001). **(B)** A similar effect is seen in the case of mechanociception (*F* = 36.099, *p* = 0.001). * *p* = 0.001, compared 3 h post CAR between 56 and 72 weeks (white bars). Data are expressed as mean ± SE.

In the adult rat group which received the CAR injection at 28 weeks and was re-infiltrated at 44 and 56 weeks, PWL and PWT were significantly increased for the 3 h response in comparison with the values obtained at 28 weeks (*F* = 28. 004, *p* = 0.001; *F* = 7.953, *p* = 0.003, respectively). In both tests, values were similar to controls for the 24 h response (Figure 5). Similarly, old rats exposed for the first time at 56 weeks of age and then re-infiltrated at 72 weeks exhibited similar results than those obtained in young and adult rats (Figure 6).

**Figure 7:**
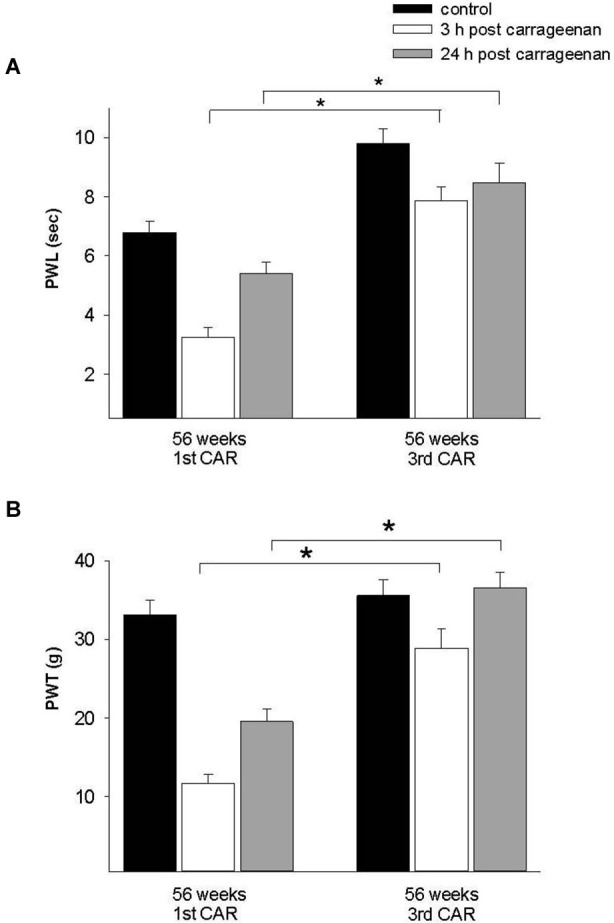
**Effect of repeated inflammation on nociceptive responses in old rats. (A)** Differences in the thermonociceptive and **(B)** mechanociceptive response in 56-week old rats which had a CAR injection for a first or third time. The first application caused a significant decrease in both responses 3 h post-injection (white bars), whereas with a third application the latency to withdrawal is increased in both tests (*F* = 28.258, *p* = 0.001; *F* = 31.965, *p* = 0.001). * *p* = 0.001. Data are expressed as mean ± SE.

To assess the pain history in old rats (56 weeks old), we compared a group exposed for the first time to the inflammatory process with a group repeatedly exposed to this stimulus. Results showed an increment in PWL and PWT at 3 h in comparison with rats exposed for the first time. At 24 h post injection, PWL and PWT values of rats exposed for the first time to the stimulus did not return to control values, whereas in the repeated exposure group, these parameters returned to control values (Figure 7).

## Discussion

This study compares antialgesic responses to thermal and mechanical stimuli between young, adult and old rats after inflammation induced by carrageenan. The reasoning behind the experimental design consisted in initially evaluating thermo and mechanociceptive responses between young (12 weeks old) and old (56 weeks old) rats exposed for the first time to the algesic procedure. Hence, any changes in the antialgesic responses measures would have only been given by the age difference. Such differences in thermal and mechanical nociception between young and old rats were not seen. The similarity in the responses is evidence not only of the operational integrity of sensory receptors associated with noxious mechanic and thermal detection but also of the functionality and conduction velocity of c and aδ fibers. In contrast to our results, Taguchi et al. (2010), using behavioral and electrophysiological techniques found that mechanical and thermal nociceptive sensory input decreased with age by deteriorating conduction velocity in mechanociceptive and nociceptive c fibers in old rats. These differences could be partially due to the age of the rats used, 56–72 vs. 127–138 weeks, in both studies, which suggests greater anatomical deterioration of pain pathways associated with age (Bergman and Ulfhake, 2002). Another difference in the present results is given by the use of a moderate inflammatory process (CAR infiltration), which generates gradual changes that are noticeable when such process is repeated as opposed to direct electrical stimulation to the nerves in which the painful threshold is reached more rapidly. Furthermore, CAR infiltration in young and old rats produces a similar inflammatory process which supports the idea that the noxious stimulus is constant throughout the experiments. This also suggests that the antialgesic alarm system is preserved at least in rats up to 72 weeks of age.

A different scenario is given when the inflammatory stimulus is repeated in the same individual throughout life, regardless of its age. The repetition of the inflammatory stimulus in young (15 and 17 weeks old), adult (48 and 56 weeks old) and old (72 week old) rats provoques an increased latency in antialgesic reflexes to thermal and mechanical stimuli. This implies a desensitization process to repeated nociceptive stimuli described as pain induced analgesia that can be explained as a plastic process that uses the inhibitory mechanisms of the diffuse noxious inhibitory controls (DNIC; Le Bars et al., 1986; López-Avila et al., 1999; Iwata et al., 2002; Kitagawa et al., 2005), especially in time windows longer than 24 h, even weeks, which is in concordance with the results presented in this study. Moreover, when old animals repeatedly stimulated are compared with animals with no pain history, we found that a desensitization process is also present. This evidences that deterioration of the anatomical elements related to the antinociceptive response is not a factor to be considered at least in rats of 56–72 weeks of age. Those old individuals exposed for the first time behave as those young ones also exposed for the first time, whereas those old individuals (56 week old) exposed for the third time present a desensitization pattern or less pain perception. This can be attributed to the repetition of the nociceptive stimulus rather than the age of the individual.

Physiological evidence using long term neuropathic and inflammatory pain models in adult rats showed that a nociceptive process can generate central changes in dopaminergic pathways. These changes are an increase in the concentration of dopamine with subsequent analgesia. There is also evidence of increased expression of inhibitory dopamine D2 receptor mRNA in the anterior cingulate and insular cortices (Coffeen et al., 2010, 2011; Ortega-Legaspi et al., 2011). Both of these changes promoted physiological antialgesia after long term painful stimulation. Indeed, these systems have not been tested in old rats but it is possible that the same mechanisms remain intact in older animals.

It has been proposed that pain perception undergoes a similar process that other sensory systems such as sight or audition when it comes to aging. We refer here to presbyopia or senile hypoacusia that can be compared to impairment of the nociceptive response propounded as “presbyalgos” by Harkins (1996). Our results indicate that the diminution in the nociceptive response is not associated with the aging process, but rather with the pain history of the individual.

We propose not only to study pain history, but the history of the individual with respect to the nociceptive phenomenon. This point of view can change pain management and the way to address the variations in the outcomes of pain studies in humans and nociception in animal models in relation to the aging process.

## Conflict of interest statement

The authors declare that the research was conducted in the absence of any commercial or financial relationships that could be construed as a potential conflict of interest.
